# A Novel Approach to Comparative RNA-Seq Does Not Support a Conserved Set of Orthologs Underlying Animal Regeneration

**DOI:** 10.1093/gbe/evae120

**Published:** 2024-06-24

**Authors:** Noémie C Sierra, Noah Olsman, Lynn Yi, Lior Pachter, Lea Goentoro, David A Gold

**Affiliations:** Department of Earth and Planetary Sciences, University of California, Davis, Davis, CA 95616, USA; Division of Biology and Biological Engineering, California Institute of Technology, Pasadena, CA 91125, USA; Division of Biology and Biological Engineering, California Institute of Technology, Pasadena, CA 91125, USA; Division of Biology and Biological Engineering, California Institute of Technology, Pasadena, CA 91125, USA; Department of Computing and Mathematical Sciences, California Institute of Technology, Pasadena, CA 91125, USA; Division of Biology and Biological Engineering, California Institute of Technology, Pasadena, CA 91125, USA; Department of Earth and Planetary Sciences, University of California, Davis, Davis, CA 95616, USA; Division of Biology and Biological Engineering, California Institute of Technology, Pasadena, CA 91125, USA

**Keywords:** regeneration, evolution, RNA-Seq

## Abstract

Molecular studies of animal regeneration typically focus on conserved genes and signaling pathways that underlie morphogenesis. To date, a holistic analysis of gene expression across animals has not been attempted, as it presents a suite of problems related to differences in experimental design and gene homology. By combining orthology analyses with a novel statistical method for testing gene enrichment across large data sets, we are able to test whether tissue regeneration across animals shares transcriptional regulation. We applied this method to a meta-analysis of six publicly available RNA-Seq data sets from diverse examples of animal regeneration. We recovered 160 conserved orthologous gene clusters, which are enriched in structural genes as opposed to those regulating morphogenesis. A breakdown of gene presence/absence provides limited support for the conservation of pathways typically implicated in regeneration, such as Wnt signaling and cell pluripotency pathways. Such pathways are only conserved if we permit large amounts of paralog switching through evolution. Overall, our analysis does not support the hypothesis that a shared set of ancestral genes underlie regeneration mechanisms in animals. After applying the same method to heat shock studies and getting similar results, we raise broader questions about the ability of comparative RNA-Seq to reveal conserved gene pathways across deep evolutionary relationships.

**Significance**

RNA-Seq could be a useful tool for identifying shared genes involved in animal tissue regeneration. We therefore developed a novel approach to compare RNA-Seq experiments with different designs and distantly related species. We ultimately find limited evidence for conserved genes, suggesting that rampant paralog switching has occurred over the course of evolution or that animal regeneration is not a conserved trait, at least at a transcriptional level.

## Introduction

Why regeneration occurs in some animals and not others remains an enigma in biology. It is well known that certain groups can readily regenerate lost tissues and body parts (e.g. planarian worms, salamanders, and cnidarians), while regeneration in others is restricted to specific organs or developmental stages (e.g. nematode worms, insects, and mammals). Animals with strong regenerative capabilities are distributed across the evolutionary tree without a clear pattern (Alvarado 2000), and even closely related species can demonstrate dramatically different capacities (Bely and Sikes 2010; Zattara et al. 2019). These observations lead to two competing evolutionary scenarios: body regeneration is either an ancient, conserved animal trait that has been lost to varying degrees across multiple lineages, or it is a derived trait that multiple lineages have converged upon independently. Resolving these competing hypotheses has profound consequences for the goals of comparative regenerative biology: are we searching for unifying principles or trying to determine how various animals deal with the universal problem of bodily damage?

While many studies focus on putative candidate genes underlying animal regeneration, a growing body of literature challenges any simplistic interpretation. Some genes and pathways commonly reoccur in studies. Wnt signaling, for example, has been shown to play a critical role in planarian worms (Sikes and Newmark 2013; Umesono et al. 2013), fish (Stoick-Cooper et al. 2007), amphibians (Lin and Slack 2008), and mammals (Bielefeld et al. 2013; Sanges et al. 2013; Takeo et al. 2013). Other studies suggest that key components of regeneration might be dissimilar across major groups. For example, a MARCKS-like protein that initiates limb regeneration in axolotl salamanders appears to be a vertebrate novelty (Sugiura et al. 2016). Regeneration in newts, a different set of amphibians, involves genes not found in the axolotl (Looso et al. 2013). Finally, genes such as the Oct4/POU5F1 regulator of stem cell pluripotency appear absent in invertebrates (Gold et al. 2014). It is unclear whether these observations represent anomalies obfuscating a conserved set of shared genes or if they hint at the true evolutionary convergence driving animal regeneration.

Whether the molecular mechanisms of regeneration are conserved across animals rests, in part, on what counts as a “conserved” (i.e. homologous) gene. Homologous genes can be subdivided into orthologs (genes related by vertical descent from a common ancestor) and paralogs (genes that arise by duplication events). Orthologs or paralogs may perform similar functions, but in evolutionary biology, common ancestry is what defines conservation. Paralogs cannot necessarily be traced back to a single gene in a last common ancestor; this means the utilization of paralogs by different species during regeneration does not necessarily support the hypothesis of a conserved ancestral function, as it may reflect evolutionary convergence achieved *after* gene duplication. Further complicating this matter, the ortholog/paralog distinction is contingent on the organisms being studied. As more distantly related species are analyzed, families of paralogous genes are often collapsed into a single orthologous clade (see supplementary fig. S1, Supplementary Material online, for an illustration of this phenomenon). Tests of molecular conservation therefore require careful consideration of the evolutionary history of genes.

The problem is compounded when using RNA-Seq technology to identify “conserved” genes between distantly related taxa undergoing similar biological processes. The first issue is a biological one: genes rarely share one-to-one homology across distantly related species. An ancestral gene might, over the course of evolution, undergo multiple, lineage-specific rounds of duplication. The second issue is technical: RNA-Seq studies have varying temporal resolutions, timescales, and depths of sequencing. When looking for significant differences in gene expression, these two issues result in a heterogeneous list of statistical tests that are problematic to compare between studies. As an example, imagine a conserved orthologous gene group, where a sea sponge has one gene sampled at three time points, while an axolotl has five genes sampled at seven time points. If all time points are compared with each other, this would result in three statistical tests for the sea sponge compared with 140 tests for the axolotl.

To address this discrepancy, we used a Lancaster *P*-value aggregation method, which provides a systematic way of collapsing multiple statistical tests for significant differential expression from multiple homologous genes into one value (Yi et al. 2018). This allows us to cluster genes into putative conserved ortholog groups (COGs) and then see which COGs are statistically enriched during the regenerative process for each species. The method takes the *P*-values generated by a differential expression analysis for a group of genes and essentially treats each as an independent significance test of the hypothesis that the broader COG is differentially expressed. Intuitively, it may be the case that no single *P*-value from a set of independent tests registers as significant; however, many borderline significant values can be aggregated to determine significance. These aggregation methods take advantage of the fact that many independent *P*-values generated by the null hypothesis should follow a uniform distribution on the interval (0, 1). Consequently, we can test the *uniformity* of the set of *P*-values to determine their likelihood of being generated from the null hypothesis. In other words, the tests of a nonsignificant COG should create a random distribution of *P*-values, while a COG with one or more significant components will statistically deviate from this distribution. Mathematically, the appropriate test statistic for uniformity can be computed from the sum of inverse cumulative distribution function with *P*-values and raw read counts as inputs. The result of this process is a table with entries corresponding to taxon–COG pairs and an associated aggregated *P*-value for each COG. supplementary fig. S2, Supplementary Material online, illustrates our approach to applying the Lancaster method to aggregate *P*-values across orthologous genes within each RNA-Seq experiment (Lancaster 1961; Yi et al. 2018). This approach allows us to make statistically honest comparisons of differential gene expression between diverse studies and elucidates what conserved genes are shared across animals during regeneration.

**Fig. 1.**
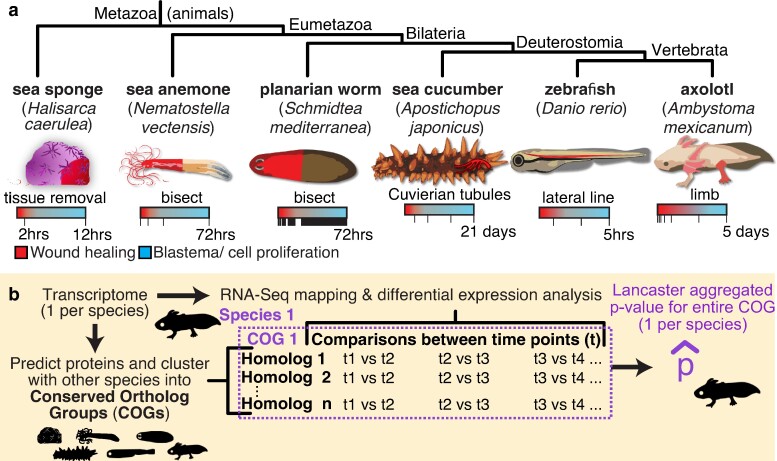
Cases of animal regeneration included in this study. a) The six animals analyzed in this paper, organized by their evolutionary relationships. The region of each organism undergoing regeneration is highlighted in red and is described underneath the image of each animal. The RNA-Seq sampling regime from each study is visualized with a bar; each time point that was sampled is represented by a notch in that bar. Despite the different absolute time ranges, the studies are comparable in that the time points span the early key stages of regeneration: starting with wound healing (red) and transitioning into blastema formation/cell proliferation (blue). b) A simplified overview of the methodology used to define deCOGS. A more detailed version is provided in supplementary fig. S2, Supplementary Material online.

In this study, we compared publicly available RNA-Seq data sets spanning wildly different organisms and structures undergoing regeneration (Fig. 1) to determine if a shared set of differentially expressed genes could be elucidated. The data sets analyzed include tissue regeneration in sea sponges (Kenny et al. 2017), oral/aboral body regeneration in sea anemones (Schaffer et al. 2016), head/tail regeneration in planarian worms (Kao et al. 2013), regeneration of “Cuvierian tubules” in the respiratory system of sea cucumbers (Sun et al. 2013), hair cell regeneration in zebrafish (Jiang et al. 2014), and limb regeneration in axolotl salamanders (Wu et al. 2013: 201). These data sets are highly divergent in their sampling regimes but cover the relevant early window between wound healing and blastema formation/cell proliferation (Fig. 1). Despite the limitations inherent in comparative RNA-Seq (considered in detail in the Discussion), this study provides a first-order analysis to clarify what is conserved in animal regeneration at a transcriptional level.

## Results

The first step was to organize all proteins from our six data sets into clusters of putative orthologs. We used OrthoFinder (Emms and Kelly 2015) to assign orthology, as this program combines amino acid sequence similarity and phylogenetic relationships to reconstruct the evolutionary history of gene families. OrthoFinder assigned 266,324 proteins across our six data sets into 16,116 COGs, 2,287 of which were present in all six data sets (see supplementary additional file S1, part 1, Supplementary Material online). These COGs were typically large, with a mean of 16.5 genes per COG. This reflected the large number of gene models in certain data sets (particularly the axolotl and zebrafish) as well as the wide evolutionary vantage taken in this study. Because we assigned orthology at the pan-animal scale, many paralogs in vertebrates or eumetazoans collapsed into a single COG in this study. As discussed later, this phenomenon is particularly important when interpreting our results. After genes were assigned to COGs, we used the Lancaster method to aggregate all *P*-values per data set per COG into one *P*-value (Yi et al. 2018). If that *P*-value met a false-discovery adjusted threshold of 0.05, we considered the COG differentially expressed for that particular data set.

**Fig. 2.**
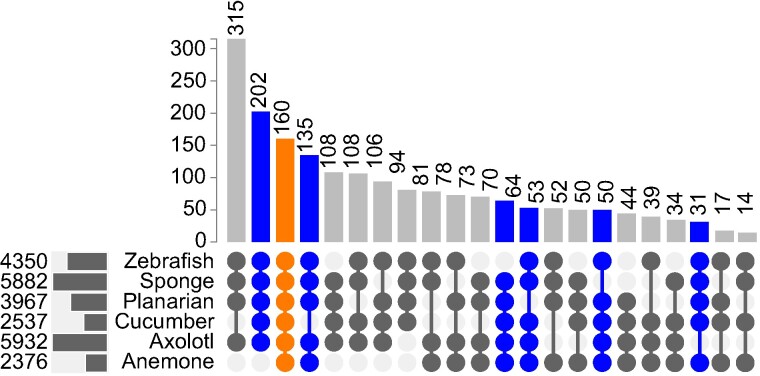
An UpSet plot demonstrating the number of overlapping deCOGs shared across all six data sets. This plot focuses on overlaps of four or more of the six data sets. The number of deCOGs common across all six cases (160) is highlighted in orange. Additional deCOGs that are recovered when individual case studies are removed are highlighted in blue. The data used to generate this figure are provided in supplementary table S2, Supplementary Material online.

To test how robust the assignment of differentially expressed COGs (deCOGs) was to variation between data sets, we examined how adding and removing data sets impacted the final number of deCOGs. Using our methodology, we recovered 160 deCOGs present in all six data sets. Removing any particular data set from the study increased the number of deCOGs shared across the remaining five data sets by an additional 31 to 202 (Fig. 2). We did not find any correlation between the quality of the RNA-Seq study and the number of additional deCOGs recovered when a data set was removed. For example, removing the sea anemone from the analysis provided the greatest increase in deCOGs, even though this data set included four RNA-Seq time points with biological replicates, as well as a well-annotated genome to work off. Conversely, the sea sponge had the poorest sampling regime, yet its removal resulted in one of the smallest gains (50 deCOGs). Instead of data set quality, the number of data set-specific deCOGs appears to be most important, as removing data sets with a small number of deCOGs (e.g. the sea cucumber and/or sea anemone) appeared to have the largest impact on overall deCOGs recovered. Ultimately, while some deCOGs could be lost due to incomplete sampling of gene expression during regeneration, our analyses do not suggest an obvious bias caused by the quality of the data sets under consideration.

A related concern to data set quality was the absence of biological replicates in some of the studies analyzed. Three of the six data sets lack biological replicates; we accepted this limitation in order to get phylogenetic diversity, though it complicated our ability to assign differentially expressed genes in those data sets (see Materials and Methods for details). To study the impact of combining data sets with and without biological replication, we looked at how many deCOGs were retained in every combination of three taxa. If we restrict our analysis to the three data sets with biological replicates (the zebrafish, anemone, and planarian), we recover 569 deCOGs. This is at the lower end compared with all combinations of three data sets (ranging from 379 to 1344 deCOGs, average = 760 deCOGs; see supplementary additional file S1, part 2.5, Supplementary Material online). The three combinations with the highest number of deCOGs all include two data sets without replicates. This suggests that our forgiving approach to dealing with data sets lacking biological replication, if anything, overestimates the true number of shared deCOGs.

**Fig. 3.**
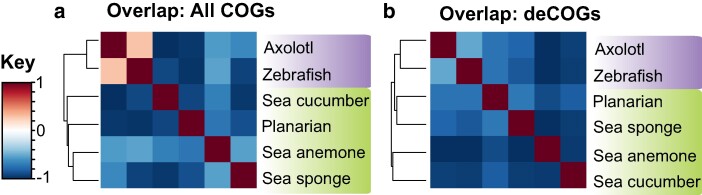
Jaccard distance matrices based on the presence/absence of COGs across taxa. a) Matrix derived from all COGs as assigned by OrthoFinder. b) The same analysis, but restricted to deCOGs The data used to generate this figure are provided in supplementary table S3, Supplementary Material online.

Following this check on the data, we proceeded with a holistic assay of COGs and discovered that the six data sets exhibit dramatically distinct gene repertoires. We used presence/absence data to construct a Jaccard distance matrix that illustrates the total number of COGs shared across data sets (Fig. 3a) and a second matrix restricted to deCOGs (Fig. 3b). The first matrix organizes the taxa on evolutionary relationships, while the second only retains the vertebrate (axolotl + zebrafish) clade. If genes expressed during regeneration represented an evolutionarily conserved network, we would anticipate the deCOG Jaccard distance matrix in Fig. 3b to show greater similarity than the full COG matrix in Fig. 3a. Instead, there appears to be even less similarity between data sets in Fig. 3b compared with Fig. 3a, although a Mantel test (Mantel 1967) suggests the two matrices are not significantly correlated. (*P* = 0.11; see supplementary additional file S1, part 2.3, Supplementary Material online). This suggests that genes expressed during regeneration are no more similar across data sets than the gene repertoires as a whole.

**Fig. 4.**
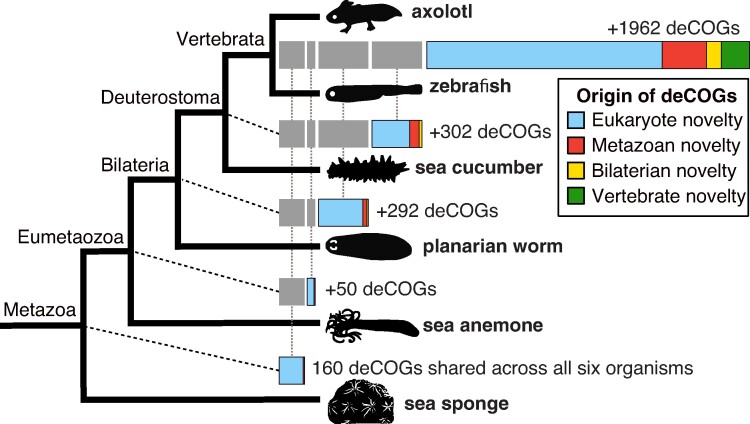
Evolutionary (phyletic) origin of deCOGs. The total number of deCOGs recovered at each node of the evolutionary tree is indicated by a bar chart to the right. Novel deCOGs at each node are broken down by their phyletic origin; for example, deCOGs that are a “bilaterian novelty” contain genes that have no significant sequence similarity to genes outside of the Bilateria. The data used to generate this figure are provided in supplementary table S4, Supplementary Material online.

One of the patterns seen in Fig. 3 is that the vertebrates (the axolotl and zebrafish) appear more similar to each other than any other combination of taxa. This raises the possibility that regeneration in vertebrates is driven by vertebrate-specific genes. To test this hypothesis, we assigned each deCOG a phyletic origin, illustrated in Fig. 4. At all nodes of the phylogeny, the majority of deCOGs can be found across diverse eukaryotes. In other words, regeneration in most animal groups does not appear to require much input from novel, animal-specific genes. While this observation holds true in the vertebrates, ∼9% of all deCOGs unique to this clade do appear to be vertebrate-specific novelties. This suggests that while the genetic control of animal regeneration is largely driven by the co-option of ancient genes, regeneration in vertebrates also requires input from genes unique to the group.

**Fig. 5.**
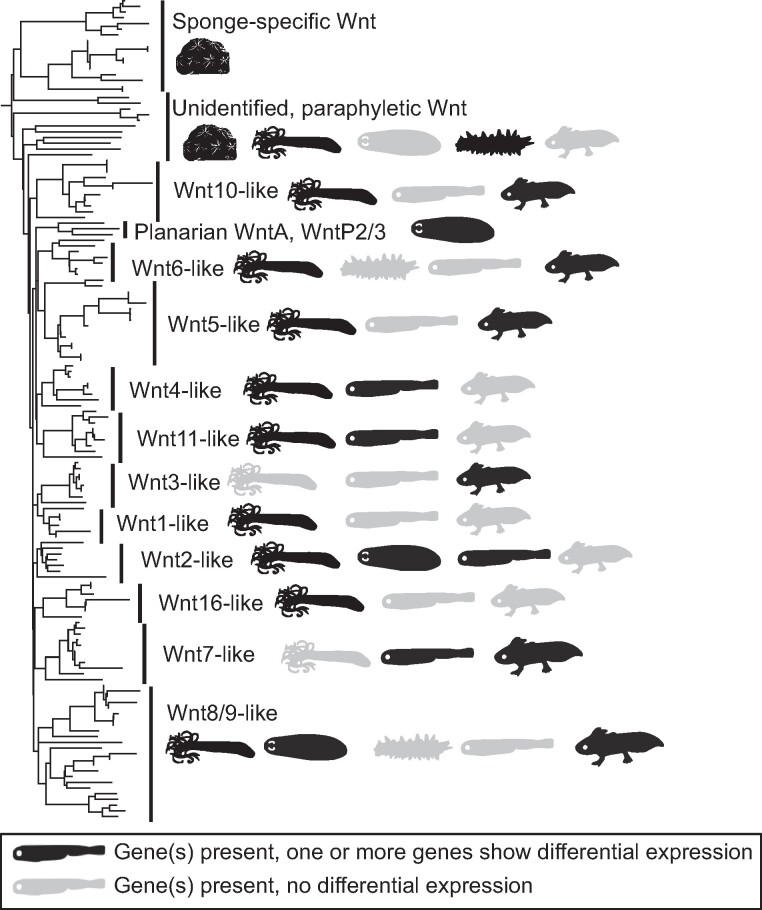
The presence of Wnt genes in the six RNA-Seq data sets analyzed (produced by OrthoFinder). Wnt genes were recovered as a single deCOG in our analysis, which can be subdivided into a minimum of 13 previously described subfamilies. The presence/absence of these subfamilies in each taxon is demonstrated by silhouettes. Gray silhouettes show the subfamily is present in the organism's transcriptome; black silhouettes show that the subfamily is present and differentially expressed in the relevant RNA-Seq study. Note that no subfamily is present and differentially expressed across all taxa. The data used to generate this figure are provided in supplementary table S5, Supplementary Material online.

After examining how the data are impacted by manipulating data sets, we next focused on the 160 deCOGs present in all six data sets. To test whether 160 deCOGs is higher than expected by chance, we performed a resampling study where we randomized the deCOGs in each data set (see Materials and Methods). The 160 deCOGs observed in our data are far greater than what is observed in our 10,000 simulation runs, where the number of shared deCOGs ranged from 8 to 44. While 160 deCOGs might therefore appear noteworthy, we note that our approach purposefully takes a generous view of what counts as “conserved.” We have, for example, ignored differences in expression direction or timing, meaning a COG is considered “conserved” if the same gene is upregulated during wound healing in one data set and downregulated in blastema formation in another. It is unlikely that such a gene actually has a conserved biological function. Moreover, the inclusion of distantly related animals in this analysis means that many large gene families have been reduced to a single COG. A good example of this latter issue comes from the Wnt family of genes, which are recovered as a single deCOG in our analysis. The gene tree produced by OrthoFinder is reprinted in Fig. 5. Our analysis suggests that sponge Wnt genes cannot be assigned to known subfamilies, resulting in all Wnts collapsing into one COG (see Borisenko et al. 2016, for similar results). Ignoring the sponge, only one of the Wnt subfamilies (Wnt8/9) is present in all organisms in our analysis, and no Wnt subfamily demonstrates differential expression across all taxa. So while Wnt genes are differentially expressed in every example of regeneration, each organism uses a different combination of paralogs. This result may not be entirely surprising, as the parts of the body undergoing regeneration in each animal are distinct, and each area of the body is patterned by different Wnt subfamilies during normal development (Krauss et al. 1992; Kusserow et al. 2005; Almuedo-Castillo et al. 2012; Borisenko et al. 2016; Auger et al. 2023). This result could therefore be interpreted as evidence that diverse Wnt paralogs can be removed and integrated into a conserved regeneration gene network (e.g. Somorjai et al. 2018) or, alternatively, that different organisms have independently integrated Wnt signaling into regeneration. Either way, this case study illustrates that a deCOG is not synonymous with a conserved gene and offers no support that one specific Wnt paralog has a conserved function in regeneration across animal evolution.

To explore the possible function of the 160 deCOGs recovered across all data sets, we used two highly cited web resources, STRING (Szklarczyk et al. 2014) and DAVID (Dennis et al. 2003), to perform functional enrichment analyses. We focused on the zebrafish for these analyses, as it represents the best-studied model organism in our data. The 160 deCOGs include 2,182 zebrafish transcripts, 554 of which could be considered differentially expressed (using the generous cutoff of an unadjusted *P* < 0.01). We compared this list of genes against the zebrafish genome to look for enriched biological pathways using the comprehensive and highly cited Kyoto Encyclopedia of Genes and Genomes (KEGG) database (see supplementary additional file S1, part 4.1, Supplementary Material online, for full results). According to STRING and DAVID analyses, the 554 differentially expressed zebrafish genes are enriched in basic cell processes, including melanogenesis, regulation of the actin cytoskeleton, phagosomes, and focal adhesion. Regarding KEGG pathways, Notch and mTOR signaling are recovered in both analyses, while Wnt and FoxO pathways are enriched in the STRING analysis. However, all of these enriched pathways are suspect, as they are primarily driven by multiple homologs from the same COG. For example, Wnt and Frizzled homologs represent 9 out of 11 genes driving “Wnt enrichment” in STRING. In “mTOR enrichment,” Wnt and Frizzled homologs make up 9 of the 15 genes in STRING and 11 out of 17 in DAVID. Similarly, “Notch enrichment” is driven by the presence of eight differentially expressed genes, seven of which are Delta/Jagged homologs. If these pathways were truly enriched in our data set, we would anticipate more genes from these pathways being differentially expressed. Rerunning the analysis on larger lists of deCOGs following the removal of individual data sets did not have a major impact on the pathways recovered (see supplementary additional file S1, parts 4.2 to 4.7, Supplementary Material online). When restricting ourselves to the three data sets with biological replicates, we do get modest gains in the number of genes involved in Wnt signaling, although 11 of the 36 genes driving enrichment are Wnt and Frizzled homologs (see supplementary additional file S1, part 4.9, Supplementary Material online). When we restricted our analysis to deCOGs shared between the vertebrates, we found a dramatic increase in the number of Wnt pathway genes represented (71 genes). Furthermore, mTOR (61 genes), FoxO (65 genes), and p53 signaling (34 genes) were also recovered as significantly enriched pathways. All of these pathways have been implicated in vertebrate regeneration (Di Giovanni et al. 2006; Tothova and Gilliland 2007; Yun et al. 2013; Martins et al. 2016). This is not simply a function of vertebrate-restricted genes being recovered, as >99% of the transcripts come from COGs present in at least one invertebrate and ∼43% of the COGs are present in all six species (see supplementary additional file S1, parts 4.10 to 4.13, Supplementary Material online). These results further support the hypothesis that a conserved regeneration network might exist across vertebrates but offer little evidence for conservation across the animals as a whole.

**Fig. 6.**
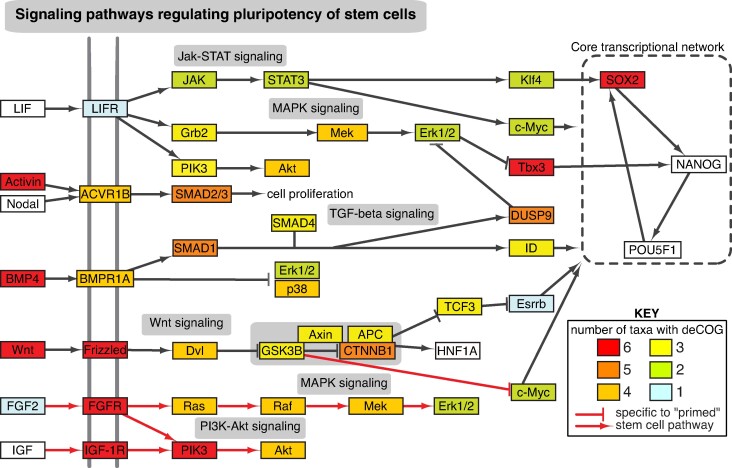
The presence of deCOGs within the stem cell pluripotency network. The network has been reproduced and simplified from KEGG pathway 04550. The color of each box indicates the number of data sets with one or more differentially expressed genes within the relevant COG. Red arrows indicate pathways that are specific to “primed” stem cells (e.g. human embryonic stem cells, human-induced pluripotent stem cells, and mouse epiblast–derived stem cells); gray arrows indicate pathways also found in “naïve” stem cells (e.g. mouse embryonic stem cells and mouse-induced pluripotent stem cells). The data used to generate this figure are provided in supplementary table S6, Supplementary Material online.

Given the long-standing interest in stem cell dynamics as a critical regulator in animal regeneration, we decided to conclude our study by exploring the representation of these pathways in our data. Figure 6 presents a simplified version of the KEGG stem cell pluripotency network (KEGG 04550), colored to indicate the number of data sets with one or more differentially expressed genes in the relevant COG. Few molecular signaling components were differentially expressed across all six data sets, and most downstream signaling targets were expressed in fewer than four data sets. Additionally, the ultimate target of these pathways—the core transcriptional network driving mammalian stem cell pluripotency (Li and Belmonte 2017)—was largely absent, with two of the genes missing from all data sets (Oct4/POU5F1 and Nanog). At first glance, some interesting signaling and receptor proteins appeared to be conserved across all six data sets. However, detailed analysis of the relevant COGs revealed that every example involved well-described paralogs being collapsed into a single pan-metazoan COG, as described previously for Wnt. Examples include “Activin” and “BMP4” being part of a single deCOG that also contains BMP2/4/5/6/8/15/16, as well as the “SOX2” deCOG, which also contains SOX1/3/9/14 (see supplementary fig. S3 and additional file S1, part 7, Supplementary Material online for details). We therefore find limited support for conserved genes in the cell pluripotency network employed in the six regeneration data sets.

### An Additional Analysis on Heat Stress Suggests the Problem of Identifying Conserved Orthologs from Comparative RNA-Seq Is Not Restricted to Regeneration

It has been several years since this study was originally posted on a preprint server. One of the reasons for the delay was an early reviewer's recommendation that we look for a “positive control,” demonstrating how the Lancaster method described here can recover conserved gene sets from pan-animal RNA-Seq data sets. After testing many data sets, we were unable to find a compelling control. An instructive example is our study of heat stress, which we anticipated would reveal COGs enriched in heat shock responses. In this project, we analyzed six data sets covering the relevant window of acute stress response to short-term heat shock in a diverse set of organisms. Our data included expression profiles of liver response of the Atlantic salmon (Shi et al. 2019), hemocyte transcriptive response in Pacific oysters (Yang et al. 2017), whole organism response in the Saharan silver ant (Willot et al. 2018), whole adult somatic tissues of a demosponge (Guzman and Conaco 2016), and a comparison of the liver transcriptome response of three breeds of commercial chickens (Lan et al. 2016). Similar to our original analysis, we were unable to recover a core set of genes governing the heat shock response. We could not find any COGs shared across all data sets and therefore focused our analysis on the comparison that produced the most results—the oyster and sponge (supplementary additional file, section 8, Supplementary Material online). Enrichment analysis of the 105 COGs found little evidence for functional conservation. DAVID Functional Annotation recovered evidence for an enrichment of the “stress response” biological process. This was driven by the differential expression of six heat shock transcripts, four of which are annotated as “heat shock protein 68.” Otherwise, all enrichment terms were related to basic cell processes, muscle/actin activity, and melanogenesis (supplementary additional file S1, section 8.3, Supplementary Material online). If we used transcript IDs from the chicken—the best studied of the five species involved—we also recovered enrichment of MAPK signaling, which was driven by 18 genes, 11 of which were calcium voltage–gated channel auxiliary subunits and 5 of which were RAS guanyl releasing proteins.

A dearth of conserved heat shock genes is not unique to our study; similar results have been found in more traditional RNA-Seq analyses, even in closely related organisms. For example, one study looking at heat stress transcriptomics in three genera of planthopper insects found only seven conserved genes, out of a total of 331 differentially expressed genes (Huang et al. 2017). In the chicken study cited earlier (Lan et al. 2016), only 9 out of 753 differentially expressed genes were conserved between all three lines (in our analysis, we combined deCOGs from all three breeds into one “chicken” data set). Similar problems in identifying conserved genes have been discussed in other biological processes, such as animal biomineralization (Gold and Vermeij 2023). Given these results, we are inclined to argue that the inability to identify conserved orthologs is a general problem in comparative RNA-Seq studies, compounded with increasing phylogenetic distance.

## Discussion

In this study, employing the Lancaster *P*-value method, we have found little evidence for a shared “core” network of orthologous genes across six RNA-Seq data sets related to regeneration. Our forgiving analysis design combined with the fact that each data set includes hundreds to thousands of differentially expressed genes makes it remarkable that so few orthologous groups were recovered. The fact that we found similar results in an analysis related to heat shock response suggests this pattern may apply more broadly in comparative RNA-Seq.

There are several ways to interpret our results. One possibility is that a conserved genetic network underlies these processes, but we failed to recover it because of insufficient RNA sampling. However, there are several arguments against this interpretation. Firstly, while it is true that the data sets included in this study had markedly different sampling regimes, they were chosen to capture overlapping, critical time frames in the regeneration processes. Secondly, removing any single taxon had minimal impact on the ortholog group content or the recovered list of differentially expressed genes (Fig. 2). Finally, the observation that phylogenetic relatedness is more predictive of gene content than the RNA sampling regime (Fig. 3a) suggests that sampling variation is insufficient to explain the differences in gene expression. So, although we cannot reject the hypothesis that deeper RNA sampling could increase the number of conserved genes, we feel confident that our results reflect a real signal in the data.

A second possibility is that a conserved genetic network underlies these processes, but we failed to recover it because the evolutionary distances between the species are too great to identify orthologs. This could contribute, in part, to the highest number of deCOGs being shared between the two vertebrates (zebrafish and axolotl), which are also the closest related pair of species in our regeneration data set. However, this is not the case in the heat shock data, where the distantly related oyster and sponge share the most deCOGs. Additionally, most vertebrate-specific deCOGs have detectable homology in the other species, which suggests it should not have a strong impact on the results. So again, we do not think our results are an artifact of the methods.

A third possibility is that regenerative abilities across the animals are a function of convergent evolution. The conserved biological processes identified across our data sets—without common transcripts driving them—reflect the common challenges multicellular organisms must address when dealing with bodily injury. This hypothesis is supported by the fact that our 160 deCOGs are far more than expected due to chance. This suggests that our results are not simply random and that the conserved pathways are those detected by enrichment analysis—namely, basic cellular processes. The lack of conservation in Wnt downstream pathway targets similarly supports this hypothesis; the presence of Wnt signaling genes across our data sets (and across studies of regeneration more broadly) could reflect the fact that there are a limited number of cell signaling pathways that animals use to pattern tissues.

A final interpretation is that an originally conserved process has been obscured over the course of evolution through developmental system drift (True and Haag 2001). In this scenario, such drift can happen when nonorthologous but functionally similar genes are recruited to perform equivalent functions (Koonin 2005a). Nonorthologous gene displacement could explain, for example, why different animals appear to use different Wnt paralogs in regeneration. When paralogs are first generated by gene duplication events, they are likely to be functionally redundant at first. This can lead to a variety of complex evolutionary dynamics, including the rapid evolution of one of the two gene copies (neofunctionalization), substitution of one paralog with another in different lineages (paralog switching), conservation of both copies (redundancy-based dosage regulation), or differences in situational deployment (subfunctionalization; Koonin 2005b; Veitia 2005). Cases such as these could open the possibility for paralog substitutions through pseudo-redundancy, where even distantly diverged paralogs may retain the ability to perform each other's functions if substituted within the relevant functional gene network. Our work adds to a growing body of evidence that nonorthologous gene displacement is commonplace in deep-time evolution (Tarashansky et al. 2021) and challenges the “ortholog conjecture” that assumes orthologs are better predictors of shared function than paralogs (Nehrt et al. 2011; Stamboulian et al. 2020).

The question, then, is how we distinguish paralog switching from evolutionary convergence. In other words, if one organism uses Wnt3 to regenerate lost tissue, and another uses Wnt4, are we gaining insight into an ancient function of Wnt genes or revealing how Wnts can be co-opted into the process of tissue repair? We conclude that distinguishing between these competing hypotheses will require the laborious reconstruction of gene regulatory networks. Similarities in regulatory binding sites and structure of network interactions, independent of paralog choice, would provide support for a common and conserved architecture in the regenerative process. An example of this comes from the careful dissection of the EGR-driven regeneration pathway in the acoel *Hofstenia* (Gehrke et al. 2019), which identified specific downstream pathways as well as regeneration-responsive chromatin regions governing the deployment of the pathway. These binding motifs and the regulatory network architecture can be specifically compared with synonymous EGR-driven regeneration networks in other organisms that are capable of similar feats of regeneration, such as sea stars and planarians.

Our results add to a growing body of literature suggesting that the molecular components of regeneration across major animal clades are largely nonorthologous. While it is possible that conserved gene regulatory networks exist, our results suggest that extensive paralog switching must have taken place and that mere comparisons of gene presence/absence from RNA-Seq experiments will prove insufficient to reveal such networks. We note that the nonhomology of animal regeneration at the transcriptional level does not negate the value of comparative studies across diverse taxa. Perhaps animal regeneration is homologous at another level of biological hierarchy (e.g. cell type regulation, tissue coordination, and organismal strategy), and the molecular logic coordinating this process evolved in an ad hoc manner across tissues and organisms. Evidence of this may come, for instance, from a recent comparison of regeneration across a sea star, planarian, and hydra, in which the authors found ample evidence of conserved gene ontologies without deeply exploring the relationships of the underlying transcripts (Cary et al. 2019). Nutrient signals have also been shown to influence regeneration activation in diverse species (Abrams et al. 2021). In this scenario, how conserved processes could be regulated by different molecular machinery would be the great challenge going forward. Alternatively, our results could signify true evolutionary convergence, in which case dozens—perhaps hundreds—of animal lineages have independently evolved solutions to bodily damage with varying degrees of success. Such a scenario puts a greater emphasis on natural selection actively driving regenerative capabilities, as opposed to such abilities being lost to genetic drift or countervailing selective forces. Given the apparent advantages of regeneration, how and why natural selection drives this trait in specific lineages would be the great challenge going forward. Detailed studies across diverse animals are needed to distinguish between these competing paradigms and determine the evolutionary history of regeneration biology.

## Materials and Methods

### Transcriptome Collection

#### Regeneration Data Set

For the axolotl (*Ambystoma mexicanum*), a transcriptome was downloaded from the Broad Institute's Axolotl Transcriptome Project (https://portals.broadinstitute.org/axolotlomics/; file: “Axolotl.Trinity.CellReports2017.fasta.gz”). For the planarian (*Schmidtea mediterranea*), a transcriptome was obtained from SmedGD (http://smedgd.stowers.org/; file: “SmedSxl Genome Annotations version 4.0 Predicted Nucleotide FASTA”). For the sea anemone (*Nematostella vectensis*), a transcriptome was downloaded from NCBI (BioProjects: PRJNA19965, PRJNA12581; file: “GCF_000209225.1_ASM20922v1_rna.fna”). For the sea cucumber (*Apostichopus japonicus*), reference isotigs were downloaded from the relevant paper (NCBI accession: GSE44995; file: “GSE44995_Reference_assembled_isotig_seq.fna.gz”; Sun et al. 2013). For the sea sponge (*Halisarca caerulea*), the transcriptome was downloaded from the Figshare link provided in the original paper (file: “Halisarca_REF_trinity.fasta.zip”). For the zebrafish (*Danio rerio*), all predicted cDNAs were downloaded from ENSEMBL release-89 (file: “GRCz10.cdna.all.fa”).

#### Heat Shock Response Data Set

The experimental transcriptome data sets for the oyster (PRJNA232944), sponge (PRJNA274004), silver ant (PRJNA419094), chicken strains (PRJEB13064), and salmon (PRJNA427772) were downloaded from the NCBI SRA Database. The SRA ID list can be found in the accessions list for each species in the associated GitHub. Total transcriptomes for mapping were retrieved in FASTA format from the NCBI “Genome” page for each of the five organisms (“transcript” downloaded in FASTA format). The genes from these transcriptomes were converted into proteins using Transdecoder (v5.0.2)^30^ and are provided in supplementary additional file 2, Supplementary Material online, on GitHub.

### Read Collection and Mapping

RNA-Seq reads were downloaded from the NCBI Sequence Read Archive (SRA) using the “fastq-dump” program in the SRA Toolkit (https://www.ncbi.nlm.nih.gov/sra). The RNA-Seq reads were aligned to the transcriptomes using HISAT-2 (Kim et al. 2015) for the regeneration data set and BOWTIE2 (Langmead and Salzberg 2012) for the heat shock data set. Both were quantified using RSEM v1.3.0 (Li and Dewey 2011). The commands used to execute RSEM are reproduced in supplementary additional file S1, part 0.1, Supplementary Material online.

### Ortholog Identification

The proteins from our species data sets were grouped into orthologous “gene sets” using the clustering algorithm OrthoFinder (Emms and Kelly 2015). All orthogroups are provided in supplementary additional file S1, part 1, Supplementary Material online. The resulting raw count matrices from RSEM were analyzed using edgeR (Robinson et al. 2010). We chose edgeR because of its ability to accept a user-defined square root dispersion value for studies that lack biological replication. The axolotl, cucumber, and sponge data sets lack biological replicates, making it impossible to estimate gene variance within samples. To deal with this shortcoming, we used edgeR to see how various values for the biological coefficient of variation (BCV) impacted the number of differentially expressed genes. According to the edgeR manual, typical values for BCV range from 0.4 for human data to 0.1 for genetically identical model organisms. We therefore tested a variety of BCV values within this space; the results are shown in supplementary fig. S4, Supplementary Material online. Multidimensional scaling plots of BCV distances for samples with biological replicates are shown in supplementary fig. S5, Supplementary Material online. We chose the lowest value for the square root dispersion (0.1), in part because this allowed for the largest number of differentially expressed genes and also because the spread of differentially expressed genes at various fold-change cutoffs behaves most similarly to data sets with biological replicates at this value (supplementary fig. S4, Supplementary Material online). edgeR was used to perform comparisons between adjacent time points. If a “wild-type” sample was included in the study, it was treated as equivalent to “time 0.” An example of the R code used to execute edgeR is reproduced in supplementary additional file S1, parts 0.2 to 0.3, Supplementary Material online. The resulting *P*-values and log count-per-million values were used for downstream aggregation of *P*-values and are also provided as supplementary additional file S3, Supplementary Material online.

### *P*-value Aggregation

Aggregation of the *P*-values produced by edgeR was based on methods described in Yi et al. (2018). The method treats each *P*-value generated from adjacent time points for a given gene as an independent test of the hypothesis that the broader COG was differentially expressed. Intuitively, it may be the case that no single *P*-value from a set of independent tests registers as significant; however, many borderline significant values can be aggregated, as in a meta-analysis, to determine significance. The aggregation methods from Yi et al. take advantage of the fact that many independent *P*-values generated by the null hypothesis should follow a uniform distribution on the interval (0, 1). Consequently, we can test the *uniformity* of the set of *P*-values to determine their likelihood of being generated from the null hypothesis. If the probability that the *P*-values as a set came from a uniform distribution is small, then we can reject the null hypothesis as having generated them. In our case, the null hypothesis corresponds to the ortholog group not being differentially expressed during regeneration for a given taxon. Mathematically, the appropriate test statistic for uniformity can be computed from the sum of inverse cumulative distribution function with *P*-values and raw read counts as inputs (see Yi et al. for details and supplementary additional file S1, part 0.5, Supplementary Material online, for Python code). The result of this process is a table with entries corresponding to taxon–ortholog group pairs and an associated aggregated *P*-value. We provide the results of this analysis in supplementary table S1, Supplementary Material online.

### False Discovery Rate Correction

Because each taxon has hundreds to thousands of distinct COGs, individual significance testing will result in many false positives. To ameliorate this, we perform the Benjamini–Hochberg procedure to adjust *P*-values for false discovery rate. The *P*-values were adjusted based on the total number of COGs such that no more than a constant fraction was likely to be false discoveries. These adjusted *P*-values were used for significance testing and resulted in a list of ortholog groups corresponding to genes that are likely to be differentially expressed during regeneration.

### Intersection Analysis

The final step was to derive a list of deCOGs shared across data sets. We originally attempted to do this by significance testing but found that numerical issues stemming from small *P*-values biased our tests such that a single *P*-value very close to 0 would yield a positive result, even if only one taxon showed strong results for that ortholog group. To avoid this problem, we instead used intersection analysis, looking at the presence/absence of deCOGs across data sets. This intersection method is less statistically rigorous but has the advantage of being robust to bias from small *P*-values.

### Correlation Plots and UpSet Plot

Overlap of COGs across taxa was visualized using correlation matrices and an Edwards Venn diagram. A binary presence/absence table for each COG was modified from the output of OrthoFinder (provided in supplementary additional file S1, part 2.1, Supplementary Material online). A second table focused on the presence/absence of deCOGs (supplementary additional file S1, part 2.2, Supplementary Material online). These tables were used to generate the Jaccard distance matrices in Fig. 3 of the main text with the corrplot R library. Commands for generating the plots are provided in supplementary additional file S1, part 2.3, Supplementary Material online. The distance matrix for all COGs and the distance matrix for deCOGs were compared using a Mantel test with 10,000 simulations. The R code for this test and the output are provided in supplementary additional file S1, part 2.3, Supplementary Material online. The table of deCOGs was used to create an UpSet plot (Lex et al. 2014).

### Resampling Study

In order to obtain the number of overlapping deCOGs that would be expected by chance, a resampling study was conducted. A number of COGs equal to the number of deCOGs observed for each data set (e.g. 5,932 COGs for the axolotl; see Fig. 2) was sampled at random from the set of all 16,116 COGs. The number of overlapping COGs across all six data sets was then calculated, and this procedure was repeated 10,000 times in order to obtain a null distribution for the number of overlapping COGs (see supplementary additional file S1, part 2.6, Supplementary Material online, for the relevant R code).

### Phylogenetic Assignment of Gene Families

Ideally, the evolutionary origin of each deCOG would be determined using a phylogenetically informed clustering analysis such as OrthoFinder. Unfortunately, taking such an approach at a eukaryote-wide scale is, for the time being, computationally prohibitive. Instead, we performed a series of BLAST queries and used sequence similarity of protein sequences to assign a phyletic origin for each COG.

Firstly, UniProt SwissProt data sets were downloaded from www.Uniprot.com using the following queries: (i) Eukaryote (nonanimal) data set, “*NOT taxonomy: “Metazoa [33208]” AND reviewed:yes*”; (ii) Early animal data set, “*taxonomy:“Metazoa [33208]” NOT taxonomy: “Bilateria [33213]” AND reviewed:yes*”; and (iii) Bilaterian invertebrate data set, “*taxonomy:“Bilateria [33213]” NOT taxonomy:“Vertebrata [7742]” AND reviewed:yes*.”

Each of these data sets was turned into a BLAST database using the *makeblastdb* command. Our query COGs were the 2,770 deCOGs present in both the zebrafish and axolotl (see Fig. 4 of the main text), which also encompassed all deCOGs at broader evolutionary scales (i.e. the deCOGs shared by all vertebrates necessarily include all deCOGs shared by deuterostomes and so on). All protein sequences from these 2,770 deCOGs were collected and formatted into a query fasta file.

With the production of our query and database files, we proceeded with an iterative process of BLAST analyses. All proteins from the 2,770 deCOGs were queried against the “Eukaryote” database using BLASTp (command: *blastp -query Query_Proteins.fasta -db Eukaryote_Dataset -outfmt 6 -evalue 10e-5 -max_target_seqs 1 -num_threads 4 -out Results.txt*). If one or query had a hit, the entire deCOG was considered a “eukaryote novelty.” Proteins in the deCOGs that did not match anything in the “Eukaryote” database were used as the query sequences for the next BLASTp analysis against the “Early animal” database. In addition, any deCOG that had no match in the “Eukaryote” database and included at least one sponge protein was automatically designated as an “animal novelty,” regardless of whether or not it had a BLAST hit in the “Early animal” database. This process was repeated until all deCOGs were assigned a phyletic origin. A summary of these results is provided in supplementary additional file S1, part 6, Supplementary Material online.

### Enrichment Analysis of deCOGs

Our comparison between all six taxa resulted in 160 deCOGs. We also examined the impact of individual taxa on the deCOG list by rerunning the analysis with one organism excluded. Zebrafish (*Danio*) gene IDs from the resulting deCOGs were collected from each analysis and are provided in supplementary additional file S1, part 3, Supplementary Material online. We restricted enrichment analysis to zebrafish genes that had at least one uncorrected (raw) *P<* 0.01 from the original edgeR analysis (supplementary additional file S1, parts 0.2 to 0.3, Supplementary Material online).

DAVID enrichment analysis was performed on the server (https://david.ncifcrf.gov). Zebrafish gene IDs were submitted using the “ENSEMBL_TRANSCRIPT_ID” identifier and a “Gene List” list type. We tested two different DAVID “Background” gene sets to compare our enriched genes against (i) all zebrafish genes in the DAVID database and (ii) zebrafish genes represented in the 2,287 COGs shared across six data sets. STRING enrichment analysis requires a list of protein IDs, so the zebrafish transcripts were converted into protein identifiers using UniProt's “Retrieve/ID mapping” function (https://www.uniprot.org/uploadlists/). The resulting IDs are provided in supplementary additional file S1, part 3, Supplementary Material online. These IDs were submitted to the STRING server for enrichment analysis (https://string-db.org). For both analyses, we restricted our study to conserved KEGG pathways. The full results of these analyses are provided in supplementary additional file S1, part 4, Supplementary Material online.

### Analysis of Gene Trees

In this paper, we examined the coverage of deCOGs in the KEGG stem cell pluripotency network (Fig. 6). For genes present in all six data sets, we went back to the OrthoFinder data to determine how gene families were organized into COGs and which genes within those COGs were differentially expressed. Species-tree corrected gene trees were collected from the OrthoFinder output. These trees were manually annotated to include gene names (based on zebrafish IDs) and whether or not genes were differentially expressed (smallest uncorrected *P* < 0.01 from edgeR output). Supplementary fig. S3, Supplementary Material online shows the gene tree for *activin* and *bmp4* constructed using this method. The tree in supplementary fig. S3, Supplementary Material online and all additional, annotated trees are provided in Newick format in supplementary additional file S1, part 7, Supplementary Material online.

## Supplementary Material

evae120_Supplementary_Data

## Data Availability

Supplementary additional file S1, Supplementary Material online and the data and code used in this study are available on GitHub at https://github.com/DavidGoldLab/2023_Comp_Regen.

## References

[evae120-B1] Abrams MJ , Tan FH, Li Y, Basinger T, Heithe ML, Sarma A, Lee IT, Condiotte ZJ, Raffiee M, Dabiri JO, et al A conserved strategy for inducing appendage regeneration in moon jellyfish, *Drosophila*, and mice. eLife. 2021:10:65092. 10.7554/eLife.65092. PMC8782573 34874003

[evae120-B2] Almuedo-Castillo M , Sureda-Gómez M, Adell T. Wnt signaling in planarians: new answers to old questions. Int J Dev Biol. 2012:56(1-3):53–65. 10.1387/ijdb.113451ma. 22450995

[evae120-B3] Alvarado AS . Regeneration in the metazoans: why does it happen? BioEssays. 2000:22(6):578–590. 10.1002/(SICI)1521-1878(200006)22:6<578::AID-BIES11>3.0.CO;2-#. 10842312

[evae120-B4] Auger NA , Medina-Feliciano JG, Quispe-Parra DJ, Colón-Marrero S, Ortiz-Zuazaga H, García-Arrarás JE. Characterization and expression of holothurian Wnt signaling genes during adult intestinal organogenesis. Genes (Basel). 2023:14(2):309. 10.3390/genes14020309. 36833237 PMC9957329

[evae120-B5] Bely AE , Sikes JM. Latent regeneration abilities persist following recent evolutionary loss in asexual annelids. Proc Natl Acad Sci U S A. 2010:107(4):1464–1469. 10.1073/pnas.0907931107. 19966282 PMC2824374

[evae120-B6] Bielefeld KA , Amini-Nik S, Alman BA. Cutaneous wound healing: recruiting developmental pathways for regeneration. Cell Mol Life Sci. 2013:70(12):2059–2081. 10.1007/s00018-012-1152-9. 23052205 PMC3663196

[evae120-B7] Borisenko I , Adamski M, Ereskovsky A, Adamska M. Surprisingly rich repertoire of Wnt genes in the demosponge *Halisarca dujardini*. BMC Evol Biol. 2016:16(1):123. 10.1186/s12862-016-0700-6. 27287511 PMC4902976

[evae120-B8] Cary GA , Wolff A, Zueva O, Pattinato J, Hinman VF. Analysis of sea star larval regeneration reveals conserved processes of whole-body regeneration across the metazoa. BMC Biol. 2019:17(1):1–19. 10.1186/s12915-019-0633-9. 30795750 PMC6385403

[evae120-B9] Dennis G , ShermanBT, HosackDA, YangJ, GaoW, LaneHC, LempickiRA. DAVID: database for annotation, visualization, and integrated discovery. Genome Biol. 2003:4(9):R60. 10.1186/gb-2003-4-9-r60.12734009

[evae120-B10] Di Giovanni S , Knights CD, Rao M, Yakovlev A, Beers J, Catania J, Avantaggiati ML, Faden AI. The tumor suppressor protein p53 is required for neurite outgrowth and axon regeneration. EMBO J. 2006:25(17):4084–4096. 10.1038/sj.emboj.7601292. 16946709 PMC1560361

[evae120-B11] Emms DM , Kelly S. OrthoFinder: solving fundamental biases in whole genome comparisons dramatically improves orthogroup inference accuracy. Genome Biol. 2015:16(1):157. 10.1186/s13059-015-0721-2. 26243257 PMC4531804

[evae120-B12] Gehrke AR , Neverett E, Luo YJ, Brandt A, Ricci L, Hulett RE, Gompers A, Ruby JG, Rokhsar DS, Reddien PW, et al Acoel genome reveals the regulatory landscape of whole-body regeneration. Science. 2019:363(6432):eaau6173. 10.1126/science.aau6173. 30872491

[evae120-B13] Gold DA , Gates RD, Jacobs DK. The early expansion and evolutionary dynamics of POU class genes. Mol Biol Evol. 2014:31(12):3136–3147. 10.1093/molbev/msu243. 25261405 PMC4245813

[evae120-B14] Gold DA , Vermeij GJ. Deep resilience: an evolutionary perspective on calcification in an age of ocean acidification. Front Physiol. 2023:14:1092321. 10.3389/fphys.2023.1092321. 36818444 PMC9935589

[evae120-B15] Guzman C , Conaco C. Gene expression dynamics accompanying the sponge thermal stress response. PLoS One. 2016:11(10):e0165368. 10.1371/journal.pone.0165368. 27788197 PMC5082814

[evae120-B16] Huang HJ , Xue J, Zhuo JC, Cheng RL, Xu HJ, Zhang CX. Comparative analysis of the transcriptional responses to low and high temperatures in three rice planthopper species. Mol Ecol. 2017:26(10):2726–2737. 10.1111/mec.14067. 28214356

[evae120-B17] Jiang L , Romero-Carvajal A, Haug JS, Seidel CW, Piotrowski T. Gene-expression analysis of hair cell regeneration in the zebrafish lateral line. Proc Natl Acad Sci U S A. 2014:111(14):E1383–E1392. 10.1073/pnas.1402898111. 24706903 PMC3986165

[evae120-B18] Kao D , Felix D, Aboobaker A. The planarian regeneration transcriptome reveals a shared but temporally shifted regulatory program between opposing head and tail scenarios. BMC Genomics. 2013:14(1):797. 10.1186/1471-2164-14-797. 24238224 PMC4046745

[evae120-B19] Kenny NJ , de Goeij JM, de Bakker DM, Whalen CG, Berezikov E, Riesgo A. Towards the identification of ancestrally shared regenerative mechanisms across the Metazoa: a transcriptomic case study in the demosponge *Halisarca caerulea*. Mar Genomics. 2017:37:135–147. 10.1016/j.margen.2017.11.001. 29198427

[evae120-B20] Kim D , Langmead B, Salzberg SL. HISAT: a fast spliced aligner with low memory requirements. Nat Methods. 2015:12(4):357–360. 10.1038/nmeth.3317. 25751142 PMC4655817

[evae120-B21] Koonin EV . Orthologs, paralogs, and evolutionary genomics. Annu Rev Genet. 2005a:39(1):309–338. 10.1146/annurev.genet.39.073003.114725. 16285863

[evae120-B22] Koonin EV . Paralogs and mutational robustness linked through transcriptional reprogramming. BioEssays. 2005b:27(9):865–868. 10.1002/bies.20296. 16108060

[evae120-B23] Krauss S , Korzh V, Fjose A, Johansen T. Expression of four zebrafish Wnt-related genes during embryogenesis. Development. 1992:116(1):249–259. 10.1242/dev.116.1.249. 1483391

[evae120-B24] Kusserow A , Pang K, Sturm C, Hrouda M, Lentfer J, Schmidt HA, Technau U, von Haeseler A, Hobmayer B, Martindale MQ, et al Unexpected complexity of the Wnt gene family in a sea anemone. Nature. 2005:433(7022):156–160. 10.1038/nature03158. 15650739

[evae120-B25] Lan X , Hsieh JC, Schmidt CJ, Zhu Q, Lamont SJ. Liver transcriptome response to hyperthermic stress in three distinct chicken lines. BMC Genomics. 2016:17(1):1–11. 10.1186/s12864-016-3291-0. 27875983 PMC5118885

[evae120-B26] Lancaster HO . The combination of probabilities: an application of orthonormal functions. Austr N Z J Stat. 1961:3:20–33. 10.1111/j.1467-842X.1961.tb00058.x.

[evae120-B27] Langmead B , Salzberg SL. Fast gapped-read alignment with Bowtie 2. Nat Methods. 2012:9(4):357–359. 10.1038/nmeth.1923. 22388286 PMC3322381

[evae120-B28] Lex A , Gehlenborg N, Strobelt H, Vuillemot R, Pfister H. Upset: visualization of intersecting sets. IEEE Trans Vis Comput Graph. 2014:20(12):1983–1992. 10.1109/TVCG.2014.2346248. 26356912 PMC4720993

[evae120-B29] Li B , Dewey CN. RSEM: accurate transcript quantification from RNA-Seq data with or without a reference genome. BMC Bioinformatics. 2011:12(1):323. 10.1186/1471-2105-12-323. 21816040 PMC3163565

[evae120-B30] Li M , Belmonte JCI. Ground rules of the pluripotency gene regulatory network. Nat Rev Genet. 2017:18(3):180–191. 10.1038/nrg.2016.156. 28045100

[evae120-B31] Lin G , Slack JM. Requirement for Wnt and FGF signaling in *Xenopus* tadpole tail regeneration. Dev Biol. 2008:316(2):323–335. 10.1016/j.ydbio.2008.01.032. 18329638

[evae120-B32] Looso M , Preussner J, Sousounis K, Bruckskotten M, Michel CS, Lignelli E, Reinhardt R, Höffner S, Krüger M, Tsonis PA, et al A de novo assembly of the newt transcriptome combined with proteomic validation identifies new protein families expressed during tissue regeneration. Genome Biol. 2013:14(2):R16. 10.1186/gb-2013-14-2-r16. 23425577 PMC4054090

[evae120-B33] Mantel N . The detection of disease clustering and a generalized regression approach. Cancer Res. 1967:27(2):209–220. 6018555

[evae120-B34] Martins R , Lithgow GJ, Link W. Long live FOXO: unraveling the role of FOXO proteins in aging and longevity. Aging Cell. 2016:15(2):196–207. 10.1111/acel.12427. 26643314 PMC4783344

[evae120-B35] Nehrt NL , Clark WT, Radivojac P, Hahn MW. Testing the ortholog conjecture with comparative functional genomic data from mammals. PLoS Comput Biol. 2011:7(6):e1002073. 10.1371/journal.pcbi.1002073. 21695233 PMC3111532

[evae120-B36] Robinson MD , McCarthy DJ, Smyth GK. edgeR: a bioconductor package for differential expression analysis of digital gene expression data. Bioinformatics. 2010:26(1):139–140. 10.1093/bioinformatics/btp616. 19910308 PMC2796818

[evae120-B37] Sanges D , Romo N, Simonte G, Di Vicino U, Tahoces AD, Fernández E, Cosma MP. Wnt/β-catenin signaling triggers neuron reprogramming and regeneration in the mouse retina. Cell Rep. 2013:4(2):271–286. 10.1016/j.celrep.2013.06.015. 23850287

[evae120-B38] Schaffer AA , Bazarsky M, Levy K, Chalifa-Caspi V, Gat U. A transcriptional time-course analysis of oral vs. aboral whole-body regeneration in the sea anemone *Nematostella vectensis*. BMC Genomics. 2016:17(1):718. 10.1186/s12864-016-3027-1. 27605362 PMC5015328

[evae120-B39] Shi KP , Dong SL, Zhou YG, Li Y, Gao QF, Sun DJ. RNA-Seq reveals temporal differences in the transcriptome response to acute heat stress in the Atlantic salmon (*Salmo salar*). Comp Biochem Physiol Part D Genomics Proteomics. 2019:30:169–178. 10.1016/j.cbd.2018.12.011. 30861459

[evae120-B40] Sikes JM , Newmark PA. Restoration of anterior regeneration in a planarian with limited regenerative ability. Nature. 2013:500(7460):77–80. 10.1038/nature12403. 23883929 PMC3812084

[evae120-B41] Somorjai IML , Martí-Solans J, Diaz-Gracia M, Nishida H, Imai KS, Escrivà H, Cañestro C, Albalat R. Wnt evolution and function shuffling in liberal and conservative chordate genomes. Genome Biol. 2018:19(1):1–17. 10.1186/s13059-018-1468-3. 30045756 PMC6060547

[evae120-B42] Stamboulian M , Guerrero RF, Hahn MW, Radivojac P. The ortholog conjecture revisited: the value of orthologs and paralogs in function prediction. Bioinformatics. 2020:36(Suppl 1):i219–i226. 10.1093/bioinformatics/btaa468. 32657391 PMC7355290

[evae120-B43] Stoick-Cooper CL , Weidinger G, Riehle KJ, Hubbert C, Major MB, Fausto N, Moon RT. Distinct Wnt signaling pathways have opposing roles in appendage regeneration. Development. 2007:134(3):479–489. 10.1242/dev.001123. 17185322

[evae120-B44] Sugiura T , Wang H, Barsacchi R, Simon A, Tanaka EM. MARCKS-like protein is an initiating molecule in axolotl appendage regeneration. Nature. 2016:531(7593):237–240. 10.1038/nature16974. 26934225 PMC4795554

[evae120-B45] Sun L , Yang H, Chen M, Ma D, Lin C. RNA-Seq reveals dynamic changes of gene expression in key stages of intestine regeneration in the sea cucumber *Apostichopus japonicus*. PLoS One. 2013:8(8):e69441. 10.1371/journal.pone.0069441. 23936330 PMC3735544

[evae120-B46] Szklarczyk D , Franceschini A, Wyder S, Forslund K, Heller D, Huerta-Cepas J, Simonovic M, Roth A, Santos A, Tsafou KP, et al STRING v10: protein–protein interaction networks, integrated over the tree of life. Nucleic Acids Res. 2014:43(D1):D447–D452. 10.1093/nar/gku1003. 25352553 PMC4383874

[evae120-B47] Takeo M , Chou WC, Sun Q, Lee W, Rabbani P, Loomis C, Taketo MM, Ito M. Wnt activation in nail epithelium couples nail growth to digit regeneration. Nature. 2013:499(7457):228–232. 10.1038/nature12214. 23760480 PMC3936678

[evae120-B48] Tarashansky AJ , Musser JM, Khariton M, Li P, Arendt D, Quake SR, Wang B. Mapping single-cell atlases throughout Metazoa unravels cell type evolution. eLife. 2021:10:e66747. 10.7554/eLife.66747. 33944782 PMC8139856

[evae120-B49] Tothova Z , Gilliland DG. Foxo transcription factors and stem cell homeostasis: insights from the hematopoietic system. Cell Stem Cell. 2007:1(2):140–152. 10.1016/j.stem.2007.07.017. 18371346

[evae120-B50] True JR , Haag ES. Developmental system drift and flexibility in evolutionary trajectories. Evol Dev. 2001:3(2):109–119. 10.1046/j.1525-142x.2001.003002109.x. 11341673

[evae120-B51] Umesono Y , Tasaki J, Nishimura Y, Hrouda M, Kawaguchi E, Yazawa S, Nishimura O, Hosoda K, Inoue T, Agata K. The molecular logic for planarian regeneration along the anterior-posterior axis. Nature. 2013:500(7460):73–76. 10.1038/nature12359. 23883928

[evae120-B52] Veitia RA . Paralogs in polyploids: one for all and all for one? Plant Cell. 2005:17(1):4–11. 10.1105/tpc.104.170130. 15632052 PMC544485

[evae120-B53] Willot Q , Mardulyn P, Defrance M, Gueydan C, Aron S. Molecular chaperoning helps safeguarding mitochondrial integrity and motor functions in the Sahara silver ant *Cataglyphis bombycina*. Sci Rep. 2018:8(1):9220. 10.1038/s41598-018-27628-2. 29907755 PMC6003908

[evae120-B54] Wu C-H , Tsai M-H, Ho C-C, Chen C-Y, Lee H-S. De novo transcriptome sequencing of axolotl blastema for identification of differentially expressed genes during limb regeneration. BMC Genomics. 2013:14(1):434. 10.1186/1471-2164-14-434. 23815514 PMC3702472

[evae120-B55] Yang C , Gao Q, Liu C, Wang L, Zhou Z, Gong C, Zhang A, Zhang H, Qiu L, Song L. The transcriptional response of the Pacific oyster *Crassostrea gigas* against acute heat stress. Fish Shellfish Immunol. 2017:68:132–143. 10.1016/j.fsi.2017.07.016. 28698121

[evae120-B56] Yi L , Pimentel H, Bray NL, Pachter L. Gene-level differential analysis at transcript-level resolution. Genome Biol. 2018:19(1):53. 10.1186/s13059-018-1419-z. 29650040 PMC5896116

[evae120-B57] Yun MH , Gates PB, Brockes JP. Regulation of p53 is critical for vertebrate limb regeneration. Proc Natl Acad Sci U S A. 2013:110(43):17392–17397. 10.1073/pnas.1310519110. 24101460 PMC3808590

[evae120-B58] Zattara EE , Fernández-Álvarez FA, Hiebert TC, Bely AE, Norenburg JL. A phylum-wide survey reveals multiple independent gains of head regeneration in Nemertea. Proc Biol Soc B. 2019:286(1898):20182524. 10.1098/rspb.2018.2524. PMC6458331 30836873

