# Supplementary material for: A Novel Approach to Comparative RNA-Seq Does Not Support a Conserved Set of Orthologs Underlying Animal Regeneration
Source: Genome Biol Evol. 2024 Jun 24;16(6):evae120. doi: 10.1093/gbe/evae120 (PMC11214158; doi:10.1093/gbe/evae120)
Supplement: evae120_Supplementary_Data [file evae120_supplementary_data.zip › Sierra_et_al-GBE-Supplementary_Figures.pdf]

SUPPLEMENTARY FIGURE LEGENDS

Figure S1. Evolutionary history and the ortholog / paralog distinction. (A) A hypothetical gene family and its evolution in two species. In this scenario, a duplication event occurred before the split of species 1 and species 2, leading to paralogs “A” and “B”. As two genes were present in the last common ancestor, the genes can be separated into two discreet conserved orthologous groups (COGs). (B) The same scenario as (A) with an additional species included. In this scenario all genes in the living species can be traced back to a single gene in the last common ancestor. From this evolutionary vantage, all 6 genes collapse into one COG.

Figure S2. Graphical Overview of methodology for identifying differentially-expressed conserved ortholog groups (deCOGs).

Figure S3. Gene tree for deCOG “OG0000461,” which includes *activin* and *bmp4* genes. Genes considered differentially expressed are labeled red.

Figure S4. Impact of BCV values on the number of differentially expressed genes in datasets lacking biological replication. The 2-fold change is noted with a grey bar; this is the standard logfold change cutoff for defining differentially expressed genes in RNA-Seq studies.

Figure S5. Multidimensional scaling plots of BCV distances between gene expression profiles for datasets containing biological replicates.

Figure S1

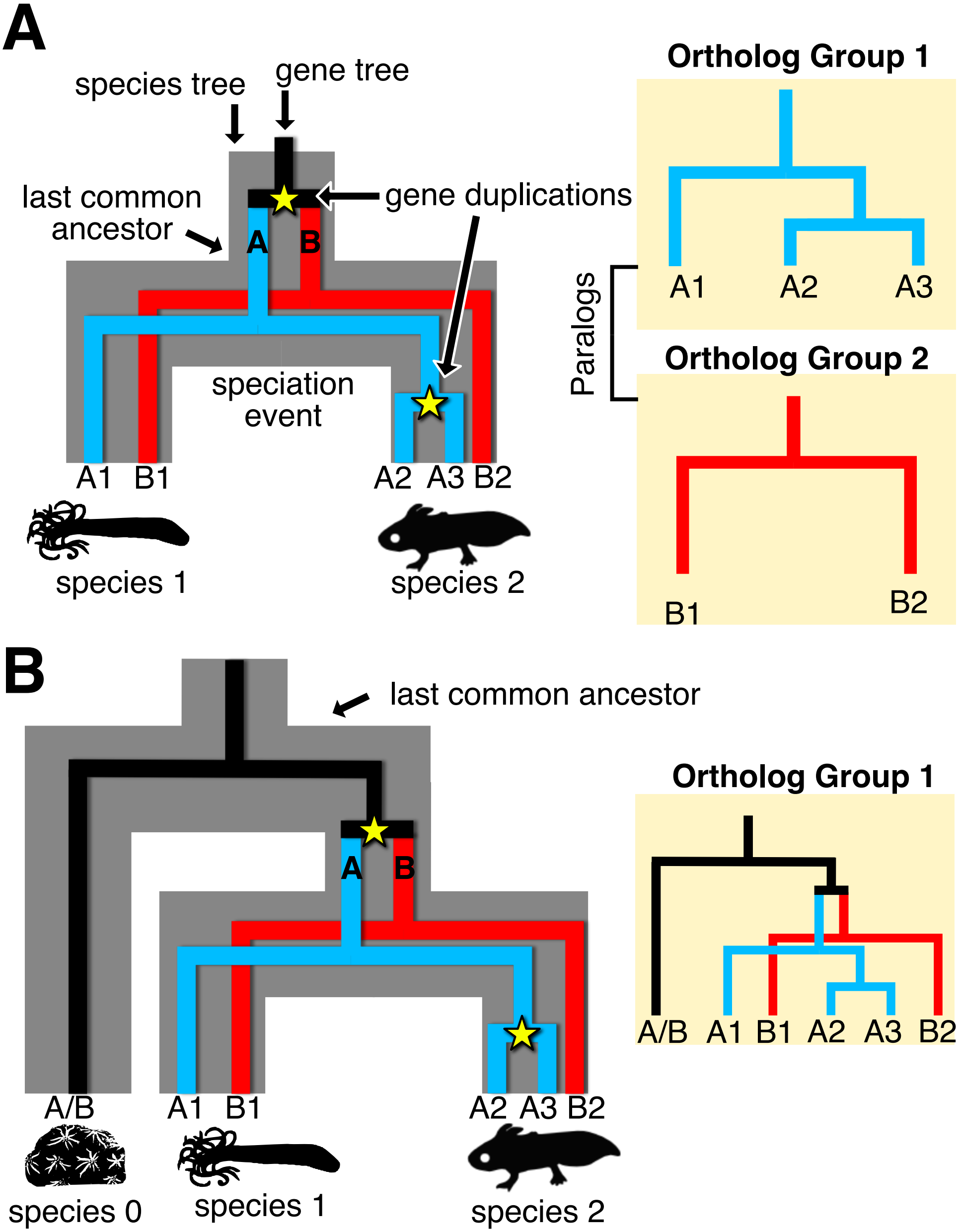
A
species tree
gene tree
last common ancestor
gene duplications
A
B
speciation event
A1 B1
A2 A3 B2
species 1
species 2
Ortholog Group 1
A1
A2
A3
Paralogs
Ortholog Group 2
B1
B2
B
last common ancestor
A
B
A/B
A1 B1
A2 A3 B2
species 0
species 1
species 2
Ortholog Group 1
A/B A1 B1 A2 A3 B2

Figure S2

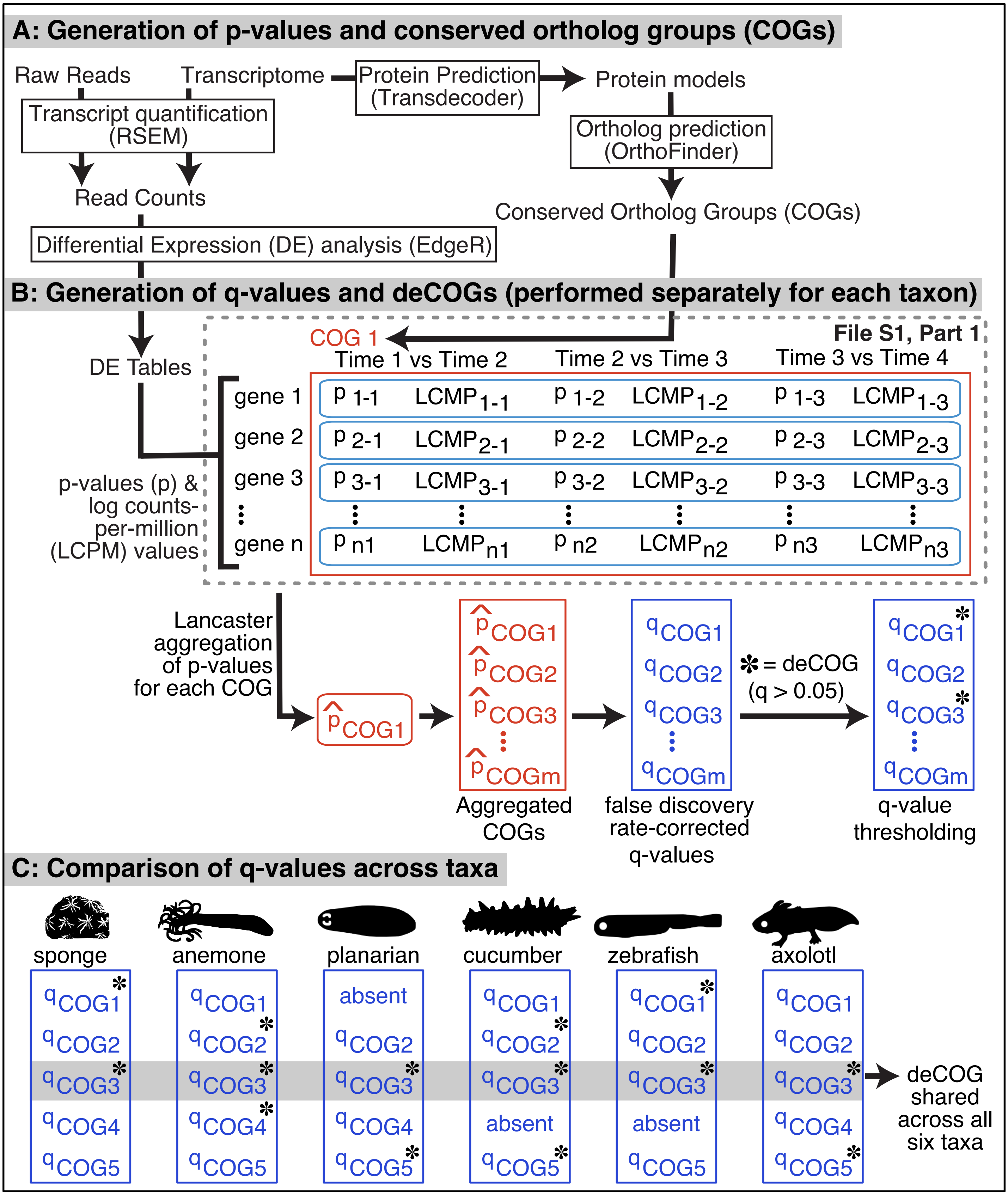
A: Generation of p-values and conserved ortholog groups (COGs)
Raw Reads
Transcriptome
Protein Prediction (Transdecoder)
Protein models
Transcript quantification (RSEM)
Ortholog prediction (OrthoFinder)
Read Counts
Conserved Ortholog Groups (COGs)
Differential Expression (DE) analysis (EdgeR)
B: Generation of q-values and deCOGs (performed separately for each taxon)
DE Tables
COG 1
File S1, Part 1
Time 1 vs Time 2
Time 2 vs Time 3
Time 3 vs Time 4
gene 1
gene 2
gene 3
gene n
p-values (p) & log counts-per-million (LCPM) values
p 1-1 LCMP 1-1 p 1-2 LCMP 1-2 p 1-3 LCMP 1-3
p 2-1 LCMP 2-1 p 2-2 LCMP 2-2 p 2-3 LCMP 2-3
p 3-1 LCMP 3-1 p 3-2 LCMP 3-2 p 3-3 LCMP 3-3
p n1 LCMP n1 p n2 LCMP n2 p n3 LCMP n3
Lancaster aggregation of p-values for each COG
pCOG1
pCOG1
pCOG2
pCOG3
pCOGm
Aggregated COGs
qCOG1
qCOG2
qCOG3
qCOGm
false discovery rate-corrected q-values
✻ = deCOG (q > 0.05)
q-value thresholding
C: Comparison of q-values across taxa
sponge
anemone
planarian
cucumber
zebrafish
axolotl
qCOG1
qCOG2
qCOG3
qCOG4
qCOG5
absent
deCOG shared across all six taxa

Figure S3

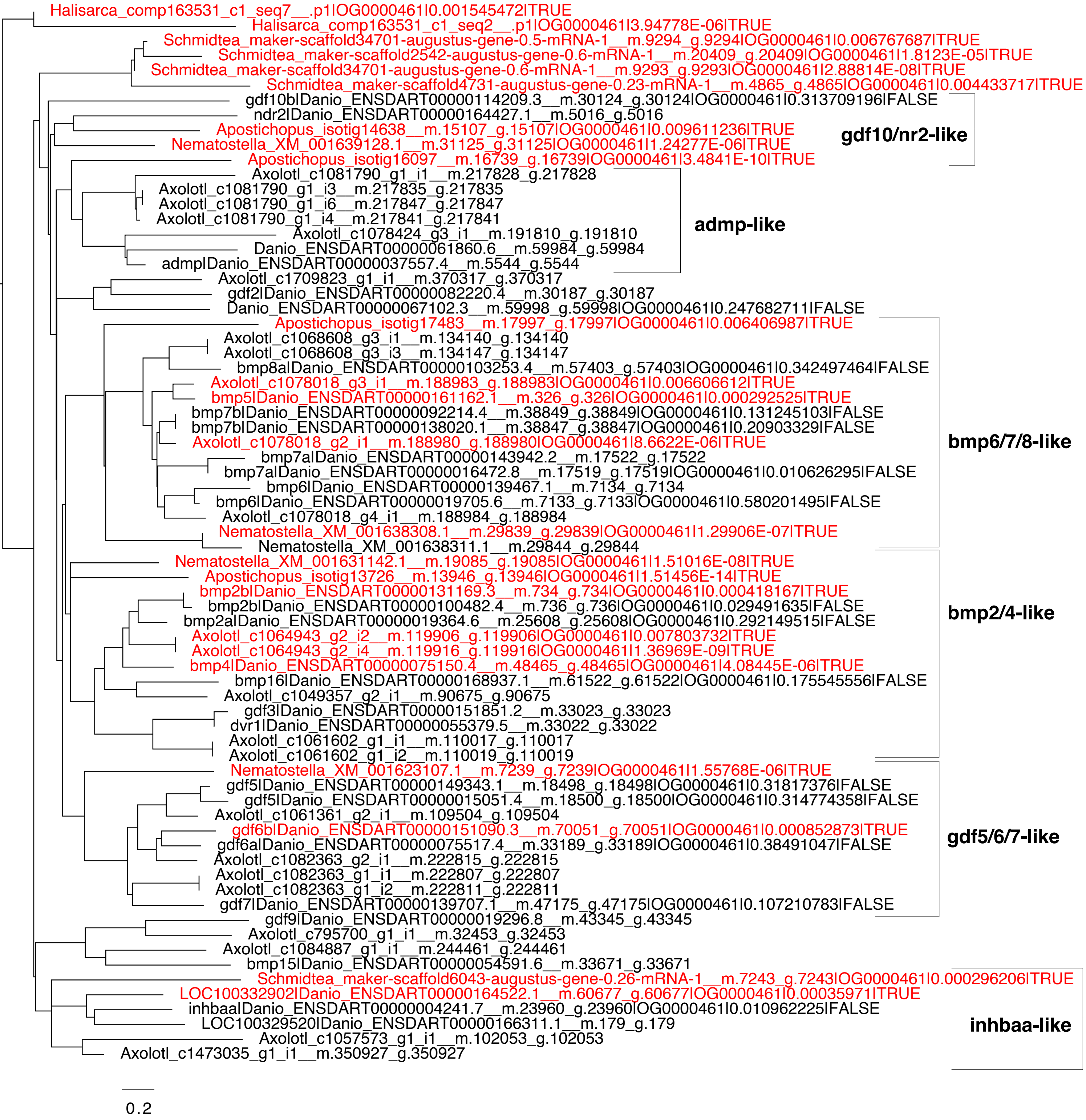

Halisarca_comp163531_c1_seq7__.p1|OG0000461|0.001545472|TRUE
Halisarca_comp163531_c1_seq2__.p1|OG0000461|3.94778E-06|TRUE
Schmidtea_maker-scaffold34701-augustus-gene-0.5-mRNA-1__m.9294_g.9294|OG0000461|0.006767687|TRUE
Schmidtea_maker-scaffold2542-augustus-gene-0.6-mRNA-1__m.20409_g.20409|OG0000461|1.8123E-05|TRUE
Schmidtea_maker-scaffold34701-augustus-gene-0.6-mRNA-1__m.9293_g.9293|OG0000461|2.88814E-08|TRUE
Schmidtea_maker-scaffold4731-augustus-gene-0.23-mRNA-1__m.4865_g.4865|OG0000461|0.004433717|TRUE
gdf10b|Danio_ENSDART00000114209.3__m.30124_g.30124|OG0000461|0.313709196|FALSE
ndr2|Danio_ENSDART00000164427.1__m.5016_g.5016
Apostichopus_isotig14638__m.15107_g.15107|OG0000461|0.009611236|TRUE
Nematostella_XM_001639128.1__m.31125_g.31125|OG0000461|1.24277E-06|TRUE
Apostichopus_isotig16097__m.16739_g.16739|OG0000461|3.4841E-10|TRUE
gdf10/nr2-like
Axolotl_c1081790_g1_i1__m.217828_g.217828
Axolotl_c1081790_g1_i3__m.217835_g.217835
Axolotl_c1081790_g1_i6__m.217847_g.217847
Axolotl_c1081790_g1_i4__m.217841_g.217841
Axolotl_c1078424_g3_i1__m.191810_g.191810
Danio_ENSDART00000061860.6__m.59984_g.59984
admp|Danio_ENSDART00000037557.4__m.5544_g.5544
admp-like
Axolotl_c1709823_g1_i1__m.370317_g.370317
gdf2|Danio_ENSDART00000082220.4__m.30187_g.30187
Danio_ENSDART00000067102.3__m.59998_g.59998|OG0000461|0.247682711|FALSE
Apostichopus_isotig17483__m.17997_g.17997|OG0000461|0.006406987|TRUE
Axolotl_c1068608_g3_i1__m.134140_g.134140
Axolotl_c1068608_g3_i3__m.134147_g.134147
bmp8a|Danio_ENSDART00000103253.4__m.57403_g.57403|OG0000461|0.342497464|FALSE
Axolotl_c1078018_g3_i1__m.188983_g.188983|OG0000461|0.006606612|TRUE
bmp5|Danio_ENSDART00000161162.1__m.326_g.326|OG0000461|0.000292525|TRUE
bmp7b|Danio_ENSDART00000092214.4__m.38849_g.38849|OG0000461|0.131245103|FALSE
bmp7b|Danio_ENSDART00000138020.1__m.38847_g.38847|OG0000461|0.20903329|FALSE
Axolotl_c1078018_g2_i1__m.188980_g.188980|OG0000461|8.6622E-06|TRUE
bmp7a|Danio_ENSDART00000143942.2__m.17522_g.17522
bmp7a|Danio_ENSDART00000016472.8__m.17519_g.17519|OG0000461|0.010626295|FALSE
bmp6|Danio_ENSDART00000139467.1__m.7134_g.7134
bmp6|Danio_ENSDART00000019705.6__m.7133_g.7133|OG0000461|0.580201495|FALSE
Axolotl_c1078018_g4_i1__m.188984_g.188984
Nematostella_XM_001638308.1__m.29839_g.29839|OG0000461|1.29906E-07|TRUE
Nematostella_XM_001638311.1__m.29844_g.29844
bmp6/7/8-like
Nematostella_XM_001631142.1__m.19085_g.19085|OG0000461|1.51016E-08|TRUE
Apostichopus_isotig13726__m.13946_g.13946|OG0000461|1.51456E-14|TRUE
bmp2b|Danio_ENSDART00000131169.3__m.734_g.734|OG0000461|0.000418167|TRUE
bmp2b|Danio_ENSDART00000100482.4__m.736_g.736|OG0000461|0.029491635|FALSE
bmp2a|Danio_ENSDART00000019364.6__m.25608_g.25608|OG0000461|0.292149515|FALSE
Axolotl_c1064943_g2_i2__m.119906_g.119906|OG0000461|0.007803732|TRUE
Axolotl_c1064943_g2_i4__m.119916_g.119916|OG0000461|1.36969E-09|TRUE
bmp4|Danio_ENSDART00000075150.4__m.48465_g.48465|OG0000461|4.08445E-06|TRUE
bmp16|Danio_ENSDART00000168937.1__m.61522_g.61522|OG0000461|0.175545556|FALSE
Axolotl_c1049357_g2_i1__m.90675_g.90675
gdf3|Danio_ENSDART00000151851.2__m.33023_g.33023
dvr1|Danio_ENSDART00000055379.5__m.33022_g.33022
Axolotl_c1061602_g1_i1__m.110017_g.110017
Axolotl_c1061602_g1_i2__m.110019_g.110019
bmp2/4-like
Nematostella_XM_001623107.1__m.7239_g.7239|OG0000461|1.55768E-06|TRUE
gdf5|Danio_ENSDART00000149343.1__m.18498_g.18498|OG0000461|0.31817376|FALSE
gdf5|Danio_ENSDART00000015051.4__m.18500_g.18500|OG0000461|0.314774358|FALSE
Axolotl_c1061361_g2_i1__m.109504_g.109504
gdf6b|Danio_ENSDART00000151090.3__m.70051_g.70051|OG0000461|0.000852873|TRUE
gdf6a|Danio_ENSDART00000075517.4__m.33189_g.33189|OG0000461|0.38491047|FALSE
Axolotl_c1082363_g2_i1__m.222815_g.222815
Axolotl_c1082363_g1_i1__m.222807_g.222807
Axolotl_c1082363_g1_i2__m.222811_g.222811
gdf7|Danio_ENSDART00000139707.1__m.47175_g.47175|OG0000461|0.107210783|FALSE
gdf5/6/7-like
gdf9|Danio_ENSDART00000019296.8__m.43345_g.43345
Axolotl_c795700_g1_i1__m.32453_g.32453
Axolotl_c1084887_g1_i1__m.244461_g.244461
bmp15|Danio_ENSDART00000054591.6__m.33671_g.33671
Schmidtea_maker-scaffold6043-augustus-gene-0.26-mRNA-1__m.7243_g.7243|OG0000461|0.000296206|TRUE
LOC100332902|Danio_ENSDART00000164522.1__m.60677_g.60677|OG0000461|0.00035971|TRUE
inhbaa|Danio_ENSDART00000004241.7__m.23960_g.23960|OG0000461|0.010962225|FALSE
LOC100329520|Danio_ENSDART00000166311.1__m.179_g.179
Axolotl_c1057573_g1_i1__m.102053_g.102053
Axolotl_c1473035_g1_i1__m.350927_g.350927
inhbaa-like
0.2

Figure S4

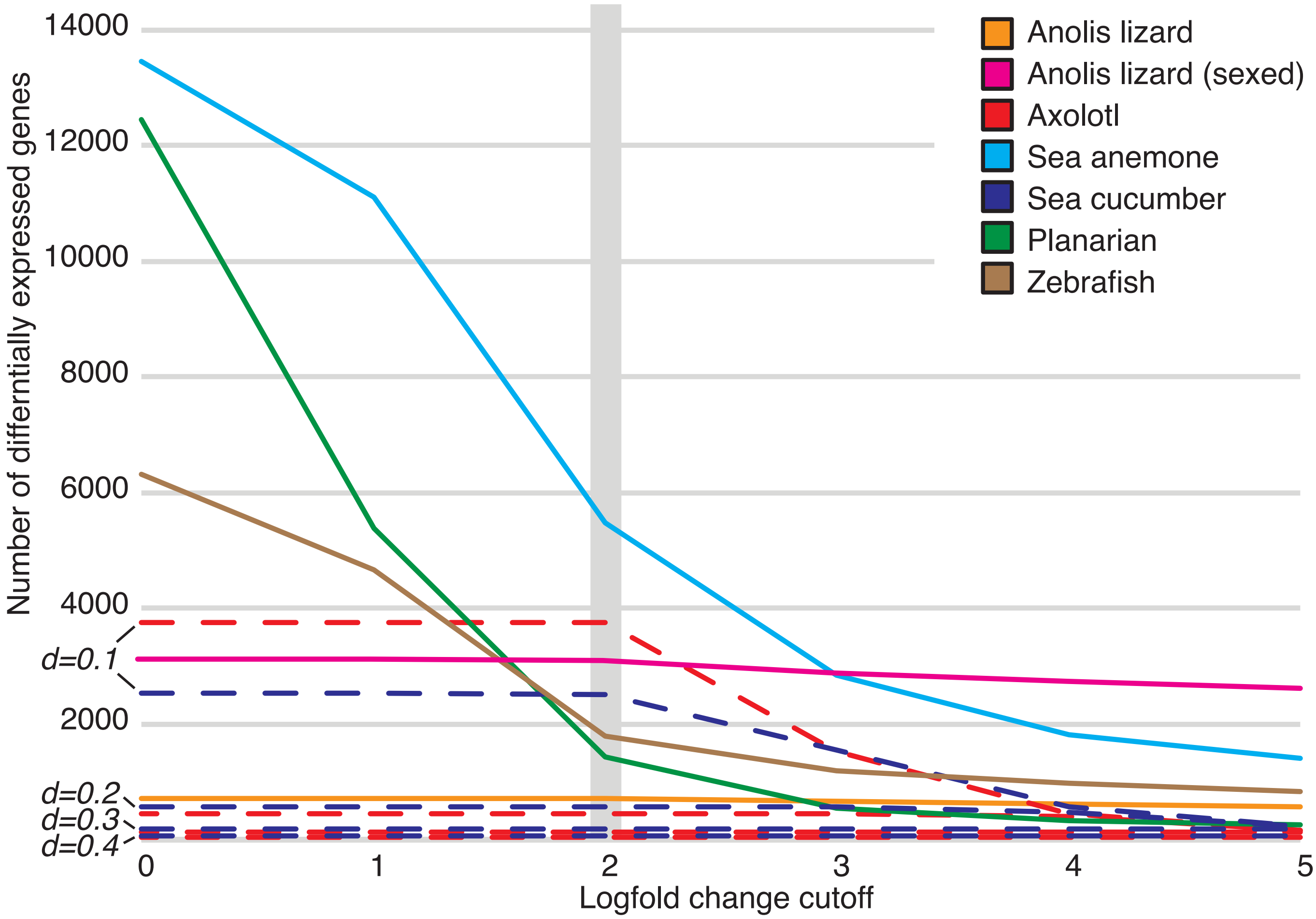
14000
12000
10000
8000
6000
4000
2000
d=0.1
d=0.2
d=0.3
d=0.4
Number of differentially expressed genes
0
1
2
3
4
5
Logfold change cutoff
Anolis lizard
Anolis lizard (sexed)
Axolotl
Sea anemone
Sea cucumber
Planarian
Zebrafish

Figure S5

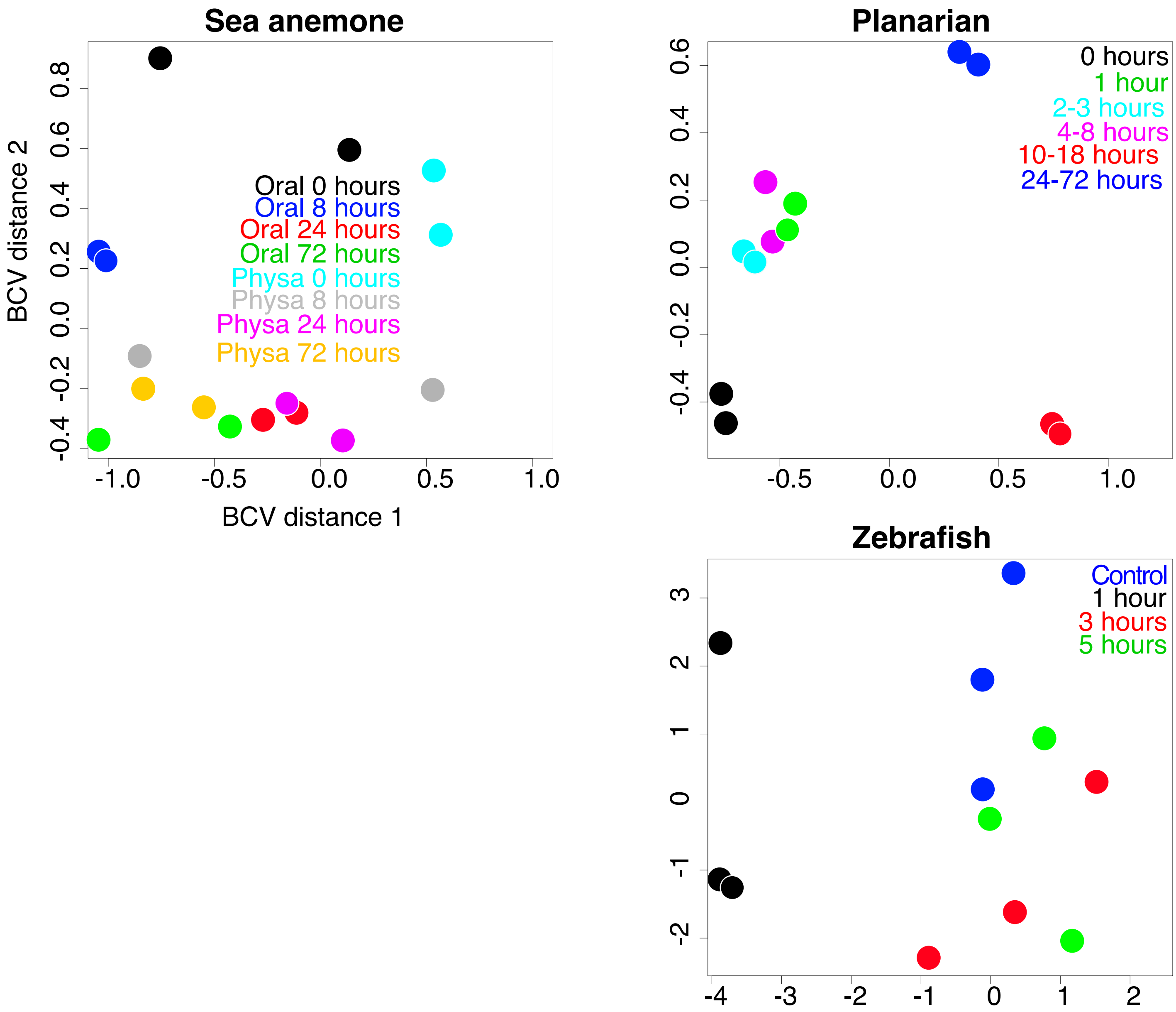
Sea anemone
BCV distance 2
BCV distance 1
Oral 0 hours
Oral 8 hours
Oral 24 hours
Oral 72 hours
Physa 0 hours
Physa 8 hours
Physa 24 hours
Physa 72 hours
-0.4
-0.2
0.0
0.2
0.4
0.6
0.8
-1.0
-0.5
0.0
0.5
1.0
Planarian
0 hours
1 hour
2-3 hours
4-8 hours
10-18 hours
24-72 hours
-0.4
-0.2
0.0
0.2
0.4
0.6
-0.5
0.0
0.5
1.0
Zebrafish
Control
1 hour
3 hours
5 hours
-2
-1
0
1
2
3
-4
-3
-2
-1
0
1
2
